# Characterization of new microsatellite markers based on the transcriptome sequencing of *Clematis finetiana*

**DOI:** 10.1186/s41065-018-0060-x

**Published:** 2018-05-15

**Authors:** Zhigao Liu, Weili Shao, Yamei Shen, Mengcheng Ji, Wenchao Chen, Ying Ye, Yongbao Shen

**Affiliations:** 1 College of Landscape Architecture, Nanjing Forestry University, Nanjing, 210037 Jiangsu People’s Republic of China; 2 College of Landscape Architecture, Zhejiang A & F University, Hangzhou 311300 Zhejiang, People’s Republic of China

**Keywords:** *Clematis finetiana*, Marker development,Transcriptome sequencing, SSRs

## Abstract

**Background:**

*Clematis* is the biggest genus in the family Ranunculaceae with about 300 species. *Clematis* is also a globally important commercial group of flowers, especially in the United States and European countries. Their petals with different colors and shapes make the genus the “Queen of the Vines”. However, the genomic information and phylogeny of *Clematis* based on existing molecular studies are limited. In this paper, new microsatellites (SSR) markers were identified from the transcriptome data of *C. finetiana* obtained using the Illumina paired-end sequencing technology.

**Results:**

Sequences on a total of 71,900 high-quality unigenes with the mean length of 865 bp were produced in this study. There were 6192unigenes annotated and classified into 49 functional sub-groups in three main ontology categories in GO (Gen Ontology) database,14,022 unigenes mapped to COGs (Clusters of Orthologous Groups) database and classified into 25 functional categories, and 21,494 unigenes obtained and divided into 128 pathways of KEGG (Kyoto Encyclopedia of Genes) Database. A total of 7532 SSRs were discovered from 6337 unigenes. We randomly tested 210 primer pairs, of which 52 primer pairs were able to generate specific products, and 19 possessed polymorphism in the 13 wild populations of six species from *Clematis*, which were used as a test material.

**Conclusions:**

The dataset of *C. finetiana* transcriptome and the identified new SSR markers will promote genetic research and breeding effort in *Clematis*.

**Electronic supplementary material:**

The online version of this article (10.1186/s41065-018-0060-x) contains supplementary material, which is available to authorized users.

## Background

There are about 300 species [1] in the genus *Clematis* L., mainly distributed in the temperate zone of the northern hemisphere [2].China has abundant germplasm resources of *Clematis*, 147 species (49%) were distributed throughout the country, especially in the southwestern area [3]. *Clematis* is the largest genus in the family Ranunculaceae, consists of typically vigorous, woody, climbing vines, and is famous for its diverse flower shapes and colors [4]; hundreds of cultivars make it the “Queen of the Vines”. *C. finetiana*is widely distributed in south China, in Zhejiang Fujian, Guangxi, Sichuan and Yunnan provinces. It is exploited and used as a medicinal plant because of triterpenoid saponins, flavonoids and many other compounds present in roots and leaves [5]. *C. finetiana* is an evergreen species, which enhances its use in balconies and for fences. It can keep growing vigorously in hot summer conditions, which makes it different from many other cultivars and species of *Clematis*. *C. finetiana* is loved by home gardening enthusiasts for its lovely white flowers and excellent heat-resistance properties. It is a good resource for heat tolerance breeding of ornamental *Clematis*.

Microsatellite (SSR) markers are an important tool for the evaluation of genetic diversity and differentiation between species and populations [6, 7]. This marker type has a generally good transferability between closely related species and it is a useful tool in genetic mapping as well [8–10]. Previously, only few molecular marker studies and DNA sequencing-based investigations have been reported on *Clematis*, including the use of ISSR primers [11], randomly amplified polymorphic DNA [12], ITS sequencing [13, 14], and single nucleotide polymorphisms (SNP) analyses of chloroplast regions accD, rps16, rpl16, trnS-trnG,atpB-rbcL, trnV-atpE and matK [15, 16]. Currently, there is a lack of SSR markers capable of effectively detecting polymorphisms in *Clematis*.

Transcriptome sequencing has been widely used for characterizing transcriptional events in a specific tissue or during a given period. It is a very useful tool also for research on non-model species that lack sequenced genome information. The high-throughput character and low cost makes RNA-seq a good choice for genetic investigations. The data resulting from transcriptome sequencing is valuable also for molecular marker development, such as microsatellite (SSR) and single nucleotide polymorphism (SNP) markers. To the best of our knowledge, very few research reports are available about the application of RNA-seqin studies on *Clematis* [17] and no information of SSR markers in *Clematis* has been published. To improve precision in genetic analyses on *Clematis*, we developed SSR markers based on the transcriptome sequencing of *C. finetiana* and utilized them to investigate inter- and intraspecific diversity and differentiation and genetic relationships among *Clematis* samples.

## Methods

### Materials and methods

#### Plant materials

Young leaves, stems and roots of *C. finetiana* were collected from three individuals. All samples were frozen in liquid nitrogen immediately and stored at − 80 °C until RNA extraction. Young leaves of wild *Clematis* germplasm of six species including112 individuals (Additional file 1) were also harvested and dried with silica gel prior to DNA extraction.

#### RNA extraction, cDNA library contraction and RNA-seq sequencing

Total RNA was isolated using the TRIzol reagent (Invitrogen, Carlsbad, CA, USA) and RNeasy® mini kit (Qiagen, Valencia, CA) according to the manufacturer’s protocol. The quality and concentration of RNA were assessed by electrophoresis on a 1.2% agarose gel and using Nanophotometer Pearl/P360 (Implen, Munich, Germany). Equal amounts of purified RNA from different tissues were pooled together for cDNA library construction and transcriptome sequencing. TransCript cDNA sample prep kit (TransGen Biotech, China) was used for cDNA library construction. Agilent 2100 Bioanaylzer (Agilent Technologies, Palo Alto, CA, USA) and1.2% agarose gel electrophoresis were used in qualification of the cDNA library. Then the library was sequenced using Illumina HiSeq™2000(Illumina).

#### Data filtering, de novo assembly and unigene function annotation

SeqPreq (https://github.com/jstjohn/SeqPrep)and sickle (https://github.com/najoshi/sickle) were used to remove sequencing adapters and trim low-quality sequences. After that, Trinity [18] software was used to assemble all clean high-quality reads. The expression level of transcripts was measured by RSEM, and the result was reported by units of TPM (transcripts per million).BLASTX was employed to annotate the function of unigene sequences using no redundant (Nr) protein database (NCBI), Swiss Prot database, KEGG (Kyoto Encyclopedia of Genes and Genomes), COG (Clusters of Orthologous Groups) and InterPro database with an E-value<10^− 5^. Blast2GO software was used for gene ontology (GO) annotation.

#### Real-time quantitative RT-PCR for verifying gene expression profiles

The equivalent mixed RNA samples (young leaves, stems and roots) from three individuals of *C. finetiana* which used for transcriptome sequencing were used as three biological replicates for the qRT-PCR experiment. And 12 genes with different expression levels were randomly selected for validating the expression results of RNA-seq sequencing. Primers (Additional file 2) were designed by Primer Premier 5(http://downloads.fyxm.net/download-now-Primer-Premier-Others-Home-&-Education-101178.html) based on the selected unigene sequences. PCR reaction mixture was consisted of 7 μl ddH_2_O, 1 μl cDNA, 10 μl 2 × SYBR® Select Master Mix (Applied Biosystems, VIC, Australia), and 1 μl each primer. qRT-PCR was carried out on ABI ViiA™ 7 Real-Time PCR system (Applied Biosystems, CA, USA) followed the cycling conditions: 95 °C for 2 min, 50 cycles at 95 °C for 10 s, 60 °C for 10 s and 72 °C for 40 s. *GAPDH* [19] was used as an internal control for normalizing the expression level of the 12 genes used for qRT-PCR testing. The specific primers of *GAPDH* were listed in (Additional file 2). The relative expression level of the selected gene was calculated via the 2^–ΔCt^.

#### SSR detection and primer design

SSR discovery was performed using the MISA software (http://pgrc.ipk-gatersleben.de/misa) with the parameters (unit size-min repeats) as follows: 1–12, 2–6, 3–5, 4–5, 5–4, 6–4. Primer pairs of each detected SSR locus were designed by Primer3 (http://bioinfo.ut.ee/primer3) with default parameters. A total of 210 primer pairs (Additional file 3) were randomly chosen and synthesized for the validation of SSR markers.

#### DNA extraction, validation of SSR markers and genetic analysis

Total genomic DNA was extracted from dry leaf tissue of 112 wild individuals from 13 populations of six *Clematis* species (Additional file 1) using the E.Z.N.A Plant DNA Mini Kit Spin Protocol (Omega Bio-tek, GA, USA) according to manufacturer’s instructions. The quality and concentration of DNA were determined by Nanophotometer Pearl/P360 (Implen, Munich, Germany).

#### PCR amplification and data analysis

PCR reactions for SSR regions were carried out in 20-μl volumes by mixing the following components: 11 μl of ddH_2_O, 2 μl of 10× buffer, 0.4 μl of 10 mM dNTP, 0.6 μl of Dynazyme II DNA polymerase (Thermo Fisher Scientific, 2 U/μl), 2 μl of genomic DNA (about 20 ng) and 2 μl (5 pmol/μl) of both primers. The PCR reactions were carried out with an initial denaturation at 94 °C for 45 s, followed by 35 cycles of 30 s at 94 °C, 30 s at SSR-specific annealing temperature (Table S2) and 40 s of elongation at 72 °C, and with a final elongation at 72 °C for 5 min. After amplification, the PCR products were diluted at 1:2–1:5 (depending on the concentration) with Milli-Q water. DNA fragments were analyzed using a capillary electrophoresis system Qsep 100DNA Analyzer (BiOptic, Taiwan, China) [20]. The observed numbers of alleles (Na), effective numbers of alleles (Ne), expected heterozygosities (He) and observed heterozygosities (Ho) were determined by Popgene1.32 [21]. Polymorphism information contents (PIC) were estimated by PowerMarker V3.25 [22] (Liu and Muse, 2005). Phylogenetic trees were constructed by PowerMarker V3.25 using the UPGMA method based on Nei’s (1979) genetic distances [23].

## Results

### RNA-seq sequencing and de novo assembly

**Fig. 1:**
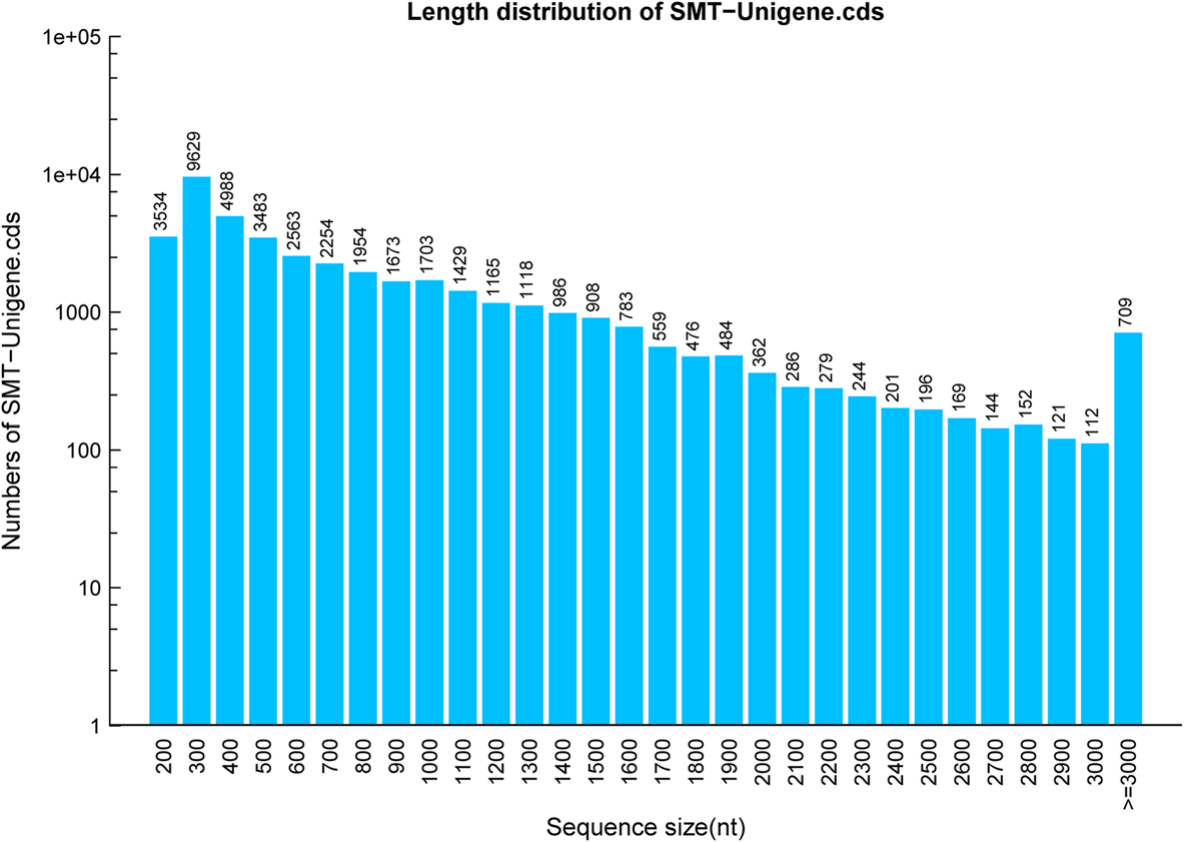
Length distribution of all unigenes obtained from the *C. finetiana* transcriptome. The x-axis indicates a different sequence size, and the y-axis indicates the unigene numbers of a specific sequence size

A total of 11.12 Gb clean bases were produced from the transcriptome sequencing of *C. finetiana*. 111.18 Mb (99.91%) clean reads with 97.56% Q20 and 93.66% Q30 were collected through data filtering. 71,900 unigenes were generated when all high-quality reads were assembled. The total length, mean length, N50 and GC content of these unigenes were 62,250, 865 bp, 1469 bp, and 42.35% (Table 1). The lengths of 21,676 (30.15%) unigenes were between 300 and 400 bp, 43,200 (60.08%) unigenes were between 400 and 2000 bp, 4760 (6.62%) unigenes were between 2000 and 3000, and 2264 (3.15%) unigenes were longer than 3000 bp (Fig. 1).

**Table 1:** Summary of transcriptome data for *C. finetiana*

Item	Number
1. Raw sequences and assembly statistics	
Total amount of clean reads(Mb)	111.18
Total amount of clean bases(Gb)	11.12
GC content percentage (%)	42.35
Clean reads proportion (%)	99.91
Total number of unigenes,	71, 900
Mean length of unigenes(bp), N50(bp), GC content(%)	865, 1469, 42.35
2.Statistics of unigene annotation	
Gene annotation against Nr (%)	36, 015 (50.09%)
Gene annotation against Swiss-Prot (%)	23, 982 (33.35%)
Gene annotation against KEGG (%)	21, 494 (29.89%)
Gene annotation against COG (%)	14, 022 (19.50%)
Gene annotation against GO (%)	6192 (8.61%)
Gene annotation against Interpro (%)	27, 004 (37.56%)
All annotated genes (%)	38, 814(53.98%)

### Functional annotation

**Fig. 2:**
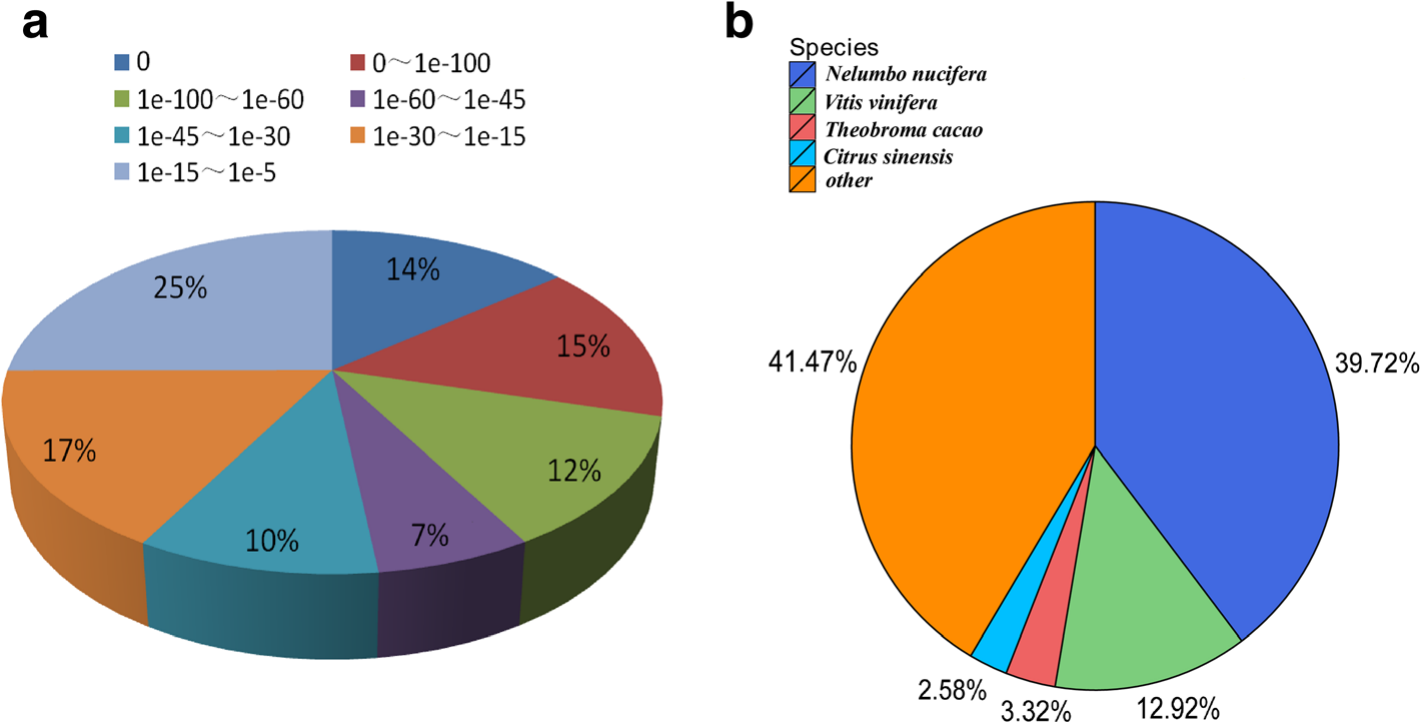
Characteristics of homology search in *C. finetiana* unigenes. (**a**) E-value distribution of the BLASTx hits against the nr database. **b** Top-hit species in similarity search of unigenes

38,814 (53.98%) out of 71,900 unigenes were annotated by at least one of the following databases: Nr, Swissprot, KEGG, COG, Interpro and GO. Nr database was the largest matched database with 36,015 unigenes (50.09% of all unigenes) annotated, followed by the Swissprot (23,982, 33.35%), KEGG (21,494, 29.89%), COG (1022, 19.50%), and Interpro (27,004, 37.56%) database (Table 1). The Nr annotation indicated that 58% of unigenes showed high homology with e-values under 1e-30, 41% of unigenes showed very high homology with e-values under 1e-60 (Fig. 2a). The top-hit species in similarity search against the Nr database included *Nelumbonucifera* (14,304, 39.72%), *Vitisvinifera* (4652, 12.92%), *Theobroma cacao* (1196, 3.32%), *Citrus sinensis* (929, 2.58%) and others (14,923, 41.47%) (Fig. 2b).

**Fig. 3:**
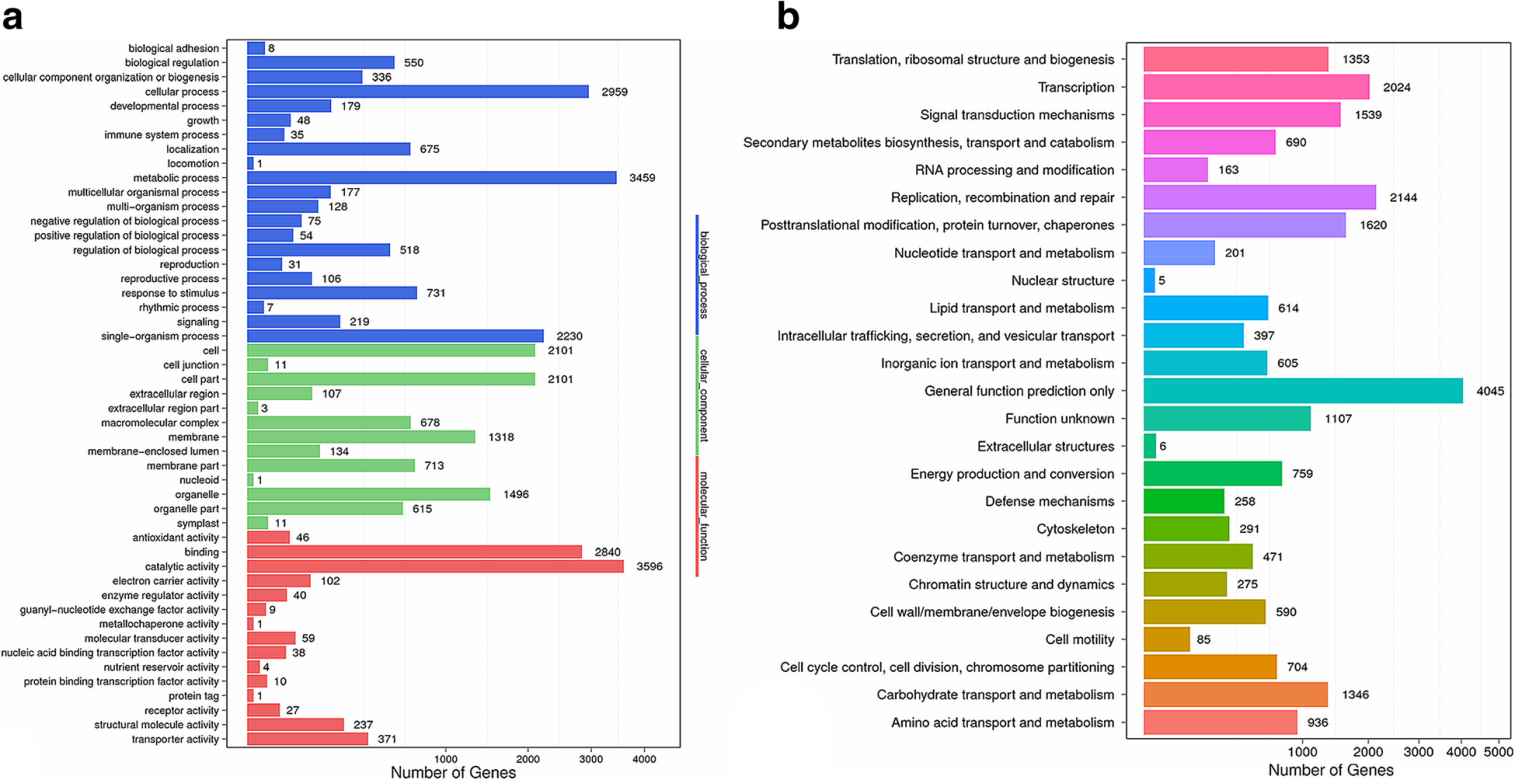
Annotation of the *C. finetiana* transcriptome. **a** GO classification of unigenes. The x-axis indicates the categories, and the y-axis indicates the number of the unigenes. **b** COG classification of unigenes. The x-axis indicates the categories, and the y-axis indicates the number of the unigenes

The GO database was employed to predict the possible function of *C. finetiana* unigenes. 6192 unigenes were annotated and classified into 49 functional sub-groups in three ontology categories, including biological processes, molecular functions and cellular components (Fig. 3a). Biological processes was the largest category with 12,526 unigenes, followed by cellular components (9289 unigenes) and molecular functions (7381 unigenes). The main groups within biological processes included metabolic processes (3495, 56.44%), cellular processes (2959,47.79%) and single-organism processes (2230, 36.01%). Cell (2101, 33.93%), cell parts (2101, 33.93%) and organelles (1496,24.16%) were the most frequent groups in the cellular components category. Catalytic activity (3596, 58.08%), binding (2840, 45.87%) and transporter activity (371, 5.99%) were the most frequent groups in the molecular functions category. According to the COG database, a total of 14,022unigenes were classified into 25 functional categories, which covered most life processes (Fig. 3b). General function was the biggest category with 4045 unigenes (6.85%), followed by the replication, recombination and repair category (2144, 3.63%) and the transcription category (2024, 3.43%).

Pathway annotations were employed to further evaluate the biological functions of the unigenes. In all, 21,494 unigenes were obtained and divided into 128 KEGG pathways (Additional file 4) which belonged to five level-1 categories, namely, cellular processes (1013, 4.71%), environmental information processing (1430, 6.65%), genetic information processing (6492, 30.20%), metabolism (13,536, 62.98) and organismal systems (1928, 8.67%). In level-2 categories, global map (5275, 24.54%), translation (2766, 12.87%), folding, sorting and degradation (1902, 8.85%), environmental adaptation (1873, 8.71%) and carbohydrate metabolism (1611, 7.50%) were the five largest categories.

### RT-qPCR validation of gene expression profiles

**Fig. 4:**
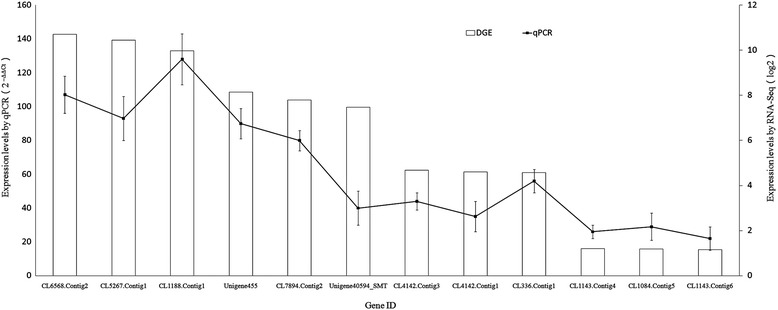
Validation of expression levels of selected genes

In order to verify the reliability of the transcriptome sequencing result, 12unigenes were randomly selected and the expression levels of them were evaluated via RT-qPCR (Fig. 4). Genes with similar expression levels in RNA-Seq (such as CL6568.Contig2, CL5267.Contig1 and CL1188.Contig1) showed a little undulating expression change in the results of RT-qPCR due to the different of sensitivity and algorithms. All tested genes presented similar change trends in RNA-Seq and qPCR experiment, indicating that the results of transcriptome sequencing were reliable.

### Development and validation of SSR markers

**Fig. 5:**
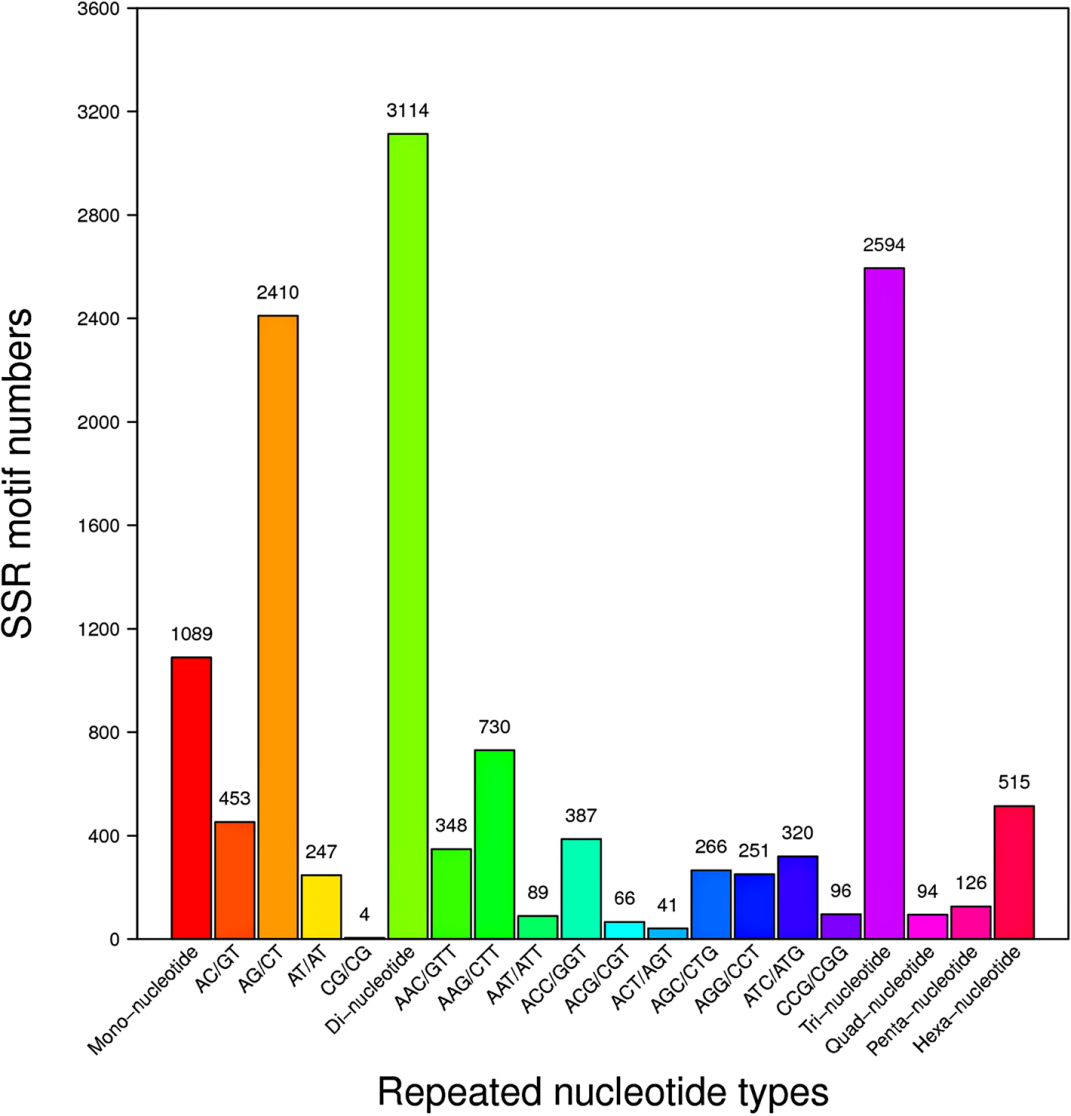
Frequency distribution of SSR repeats types. Motif types of di-nucleotides and tri-nucleotides are represented. The x-axis indicates the categories, and the y-axis indicates the number of the unigenes

In this study, MISA perl script (http://pgrc.ipk-gatersleben.de/misa) was used to detect microsatellites in unigene sequences. A total of 7532 SSRs were recognized from 6337 unigenes (Table 2). Di-nucleotides were the most abundant type (3114, 41.34%), followed by tri-nucleotides (2594, 34.44%) hexa-nucleotides (515, 6.84%), penta-nucleotides (126, 1.67%) and tetra-nucleotides (94, 1.25%). AG/CT (2410, 37.40%) was the most abundant motif type, followed by AAG/CTT (730, 11.33%), AC/GT (453, 7.03%), ACC/GGT (387, 6.01%), AAC/GTT (348, 5.04%), ATC/ATG (320, 4.97%) and others (Fig. 5). The number of repeat motifs of SSR loci (except mono-nucleotides) ranged from 4 to 28, SSRs with six motif repeats were the most common type (1715, 26.62%), followed by five (1537, 23.68%), seven (947, 14.70%) and eight repeats (659, 10.23%) types, respectively (Additional file 5).

**Table 2:** Summary of microsatellite data of *C. finetiana* transcriptome

Item	Number
Total number of sequences examined	71, 900
Total size of examined sequences (bp)	62, 250,256
Total number of identified SSRs	7532
Number of SSR containing sequences	6337
Number of sequences containing more than 1 SSR	961
Number of SSRs present in compound formation^a^	568

^a^The SSR locus containing at least 2 repeat motifs

In all, 210 SSR sites (Additional file 3) were randomly selected for primer designing. Among them, 52 (24.76%) primer pairs generated expected specific products, 38 (18.10%) primer pairs amplified PCR products much smaller or larger than the expect size, while the other 120 (57.14%) primer pairs did not amplify at all. The other five species (*C. brevicaudata, C. apiifolia*, *C. uncinata*, *C. lasiandra* and *C. henryi*) were assessed to evaluate the transferability of the 52 SSR well working markers. The results showed that 19 loci were polymorphic, and a total of 135 alleles were discovered at those 19 SSR loci among the samples including 112 accessions from 13 populations of 6 *Clematis* species. The allele sizes were around the predicted allele sizes (Additional file 6; Additional file 7). The allele numbers per locus (Na) rangedfrom 3 to 11 (average 7.11), the effective number of alleles (Ne) ranged from 1.80 to 9.65 (average 4.44). The ranges of expected homozygosity (Ho) and expected heterozygosity (He) were from 0.10 to 0.57(average 0.25) and 0.43 to 0.86 (average 0.75), respectively. The polymorphism information contents (PIC) per locus ranged from 0.41 to 0.88 (average 0.72), for the whole dataset (Additional file 8).

**Fig. 6:**
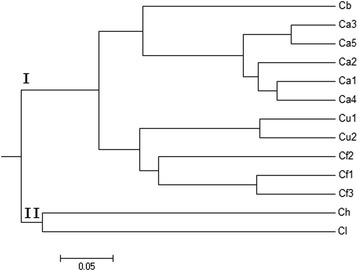
Phylogenetic tree of 13 populations from 6 *Clematis* species based on 19 SSR loci and utilizing Nei’s genetic distances. The population abbreviations are the same as those in Additional file 1

The UPGMA tree (Fig. 6) was constructed based on Nei’s genetic distances. Two main clusters were generated. Different populations from *C. apiifolia, C.finetiana* and *C. uncinata* were grouped together, respectively. *C. lasiandra* and *C. henryi* belonged to group II and the other 4 species were distributed into another bigger group I, in which *C. brevicaudata* and *C. apiifolia* were closely related, and *C. uncinata* and *C.finetiana* were grouped together.

## Discussion

*Clematis* cultivars are well known for their diverse flower types and colors; especially the large flowered hybrids make *Clematis* one of the most popular flowers. However, only few molecular marker-based approaches have been used to assist breeding of ornamental *Clematis*. Inter-simple sequence repeat markers (ISSR) have been used to fingerprint 32 vining cultivars and five no-vining species for assessing genetic relationships and cultivar identification [11]. Random amplified polymorphic DNA (RAPD) has been used to confirm the identity of *Clematis* hybrids [12]. There are several earlier reports about the systematic classification and phylogeny research on *Clematis*, which are mainly based on phenotypic characteristics, such as the shape and length of sepals, the indumentum of filaments, the structure of pollen and plant morphology [2, 3, 24, 25]. A few reports have been published about the interspecific relationships among *Clematis* based on molecular markers [11, 14, 26, 27], but such research is still limited due to the lack of genomic information of *Clematis*. In this study, the transcriptome of *C. finetiana* was sequenced in order to obtain genomic data and to develop SSR markers.

In the present study, a total of 71,900 unigenes was obtained via transcriptome sequencing with an average length of 865 bp. The average length of sequenced *C. finetiana* unigenes was longer compared to recently released transcriptome sequencing results on *Eucommia ulmoides* (645 bp) [28], *Vigna mungo* (443 bp) [29] and *Prunus sibirica* (652 bp) [30]. As a result of BLAST searches, a total of 38,814 unigenes (53.98%) were annotated by Nr, Swiss-Prot, COG, GO, Interpro and KEGG databases, while about 33,086 unigenes (46.02%) were not annotated by any of these six databases, thus indicating that some unigenes may be particular to *Clematis.* Transcriptome sequencing is also useful tool for the development of SSR markers, and there are previous reports about the development of SSRs in plants through transcriptome sequencing [31–34].

Microsatelite markers are co-dominant, highly polymorphic and easily reproducible [35]. They are important tools to examine genetic diversity, the assessment of genetic relationships and population genetic structure in plants [26]. The application of SSR markers in the study of *Clematis* has been limited due to the expenses and time-consuming work when developing markers by traditional methods. Some other molecular marker systems have been applied to studies on *Clematis*. ITS sequences have been analyzed to provide molecular evidence for the identification of 14 medicinal *Clematis* species [14, 26]. The sequences of chloroplast DNA (atpB-rbcL spacer region, matK, trnK, trnL intron, andtrnL-trnF spacer region) and the nuclear actin I intron have been used for the analysis of phylogenetic relationships within the genus *Clematis*. The result showed that the taxonomic status of several species was not consistent with previous interspecific classifications based on morphology [15, 36]. However, none of them have utilized SSR markers.

In this study, a high quantity of high-quality transcriptome sequences was obtained, which could be used to develop SSR markers for *Clematis*. A total of 7532 SSRs were recognized from 6337 unigenes. These SSRs could be divided into five categories based on the number of bases from two to six, di-nucleotides and tri-nucleotides being the most abundant types, which is in concord with previous studies [30, 37]. Furthermore, AG/CT and AAG/CTT were the most abundant motif typesamong-nucleotides and tri-nucleotides, similarly as previously reported [38]. Tetr-nucleotide and penta-nucleotide motifs of SSRs are usually less polymorphism in coding sequences region. Nevertheless, the two types were generally included in the list of screening primers [29-31]. In the present study, SSR primers of *Clematis* were screened for the first time. In consideration of the comprehensiveness of the experiment, a total of 210 primer pairs from all five SSR types (including tetr-nucleotide and penta- nucleotide motifs) were randomly chosen. We successfully developed 52 SSR markers, which gave clear amplification products and showed good transferability among different species of *Clematis*. Within the genus, 19 markers were polymorphic, and all except one of these polymorphic loci showed the presence of a high level of polymorphism with PIC values above 0.60. Several studies have indicated that tetra-nucleotide SSRs have high polymorphism rates [39]. In the present study, both two tetra-nucleotide SSRs showed high polymorphism, of then‘4–11’was the most informative tetra-nucleotide SSR with the PIC value of 0.88. Hexa-nucleotides (9, 56.25%) were the most abundant type among the 19 polymorphic SSRs, followed by tri-nucleotides (4, 21.05%) and di-nucleotides (3, 15.79%). This observation was similar to the result of SSR development in the rubber tree [40]. All amplified fragments resulting from the 19 primer pairs were around the expected size and showing that the detected polymorphism resulted from the variation in the number of SSR repeats.

Microsatellite markers have been proved to be effective tool in the evaluation of interspecific genetic diversity and phylogenetic relationship. The phylogenetic tree constructed based on SSR data showed that *C. brevicaudata* and *C. apiifolia* belong to the same group; *C. uncinata* and *C.finetiana* are closely related. These results are similar to those of Xie et al., who used nuclear ITS and three plastid regions in the phylogentic analyses of *Clematis* [13]. This result demonstrated the effectiveness in analyzing *Clematis* genetic relationships and confirming the potential value of the transcriptome database for the development of new SSR markers. The newly developed polymorphic SSR markers can be applied into population genetic and phylogenetic studies, species and hybrid identification, and possibly also to marker-assisted breeding in *Clematis*.

## Conclusion

The present study reported the functional characterization of transcriptome sequences of *Clematis* and development of new SSR markers, which are applicable to many kinds of studies in *Clematis*. To the best of our knowledge, this is the first report on the development and use of any kind microsatellite markers in the genus *Clematis*. They can be used in further studies on the genetic diversity, population genetics and phylogeography of *Clematis*, and they can assist the breeding of new ornamental cultivars.

## Additional files

Additional file 1: Sampling information of the six Clematis species. (XLSX 10 kb)

Additional file 2: Used primers for qRT-PCR experiments. (XLSX 12 kb)

Additional file 3: Details of 210 selected SSR markers used for polymorphism validation. (XLSX 34 kb)

Additional file 4: The KEGG pathway annotations. (XLSX 10 kb)

Additional file 5: The frequency of classified SSR repeat types. (XLSX 24 kb)

Additional file 6: Detected allele sizes of 19 SSR loci in different Clematis species. (XLSX 12 kb)

Additional file 7: The product sizes of the amplified locus ‘6-73’ in 4 Clematis samples determined by Qsep 100 DNA Analyzer. (PNG 136 kb)

Additional file 8: Characteristics of 19 polymorphic SSR loci. (XLSX 11 kb)

## Abbreviations

COG: Clusters of Orthologous Groups
GO: Gene Ontology
KEGG: Kyoto Encyclopedia of Genes and Genomes
Nr: Non-redundant
SSR: Simple sequence repeat marker
