# Supplementary figures and images for: Characterization of new microsatellite markers based on the transcriptome sequencing of Clematis finetiana
Source: Hereditas. 2018 May 15;155:23. doi: 10.1186/s41065-018-0060-x (PMC5952850; doi:10.1186/s41065-018-0060-x)

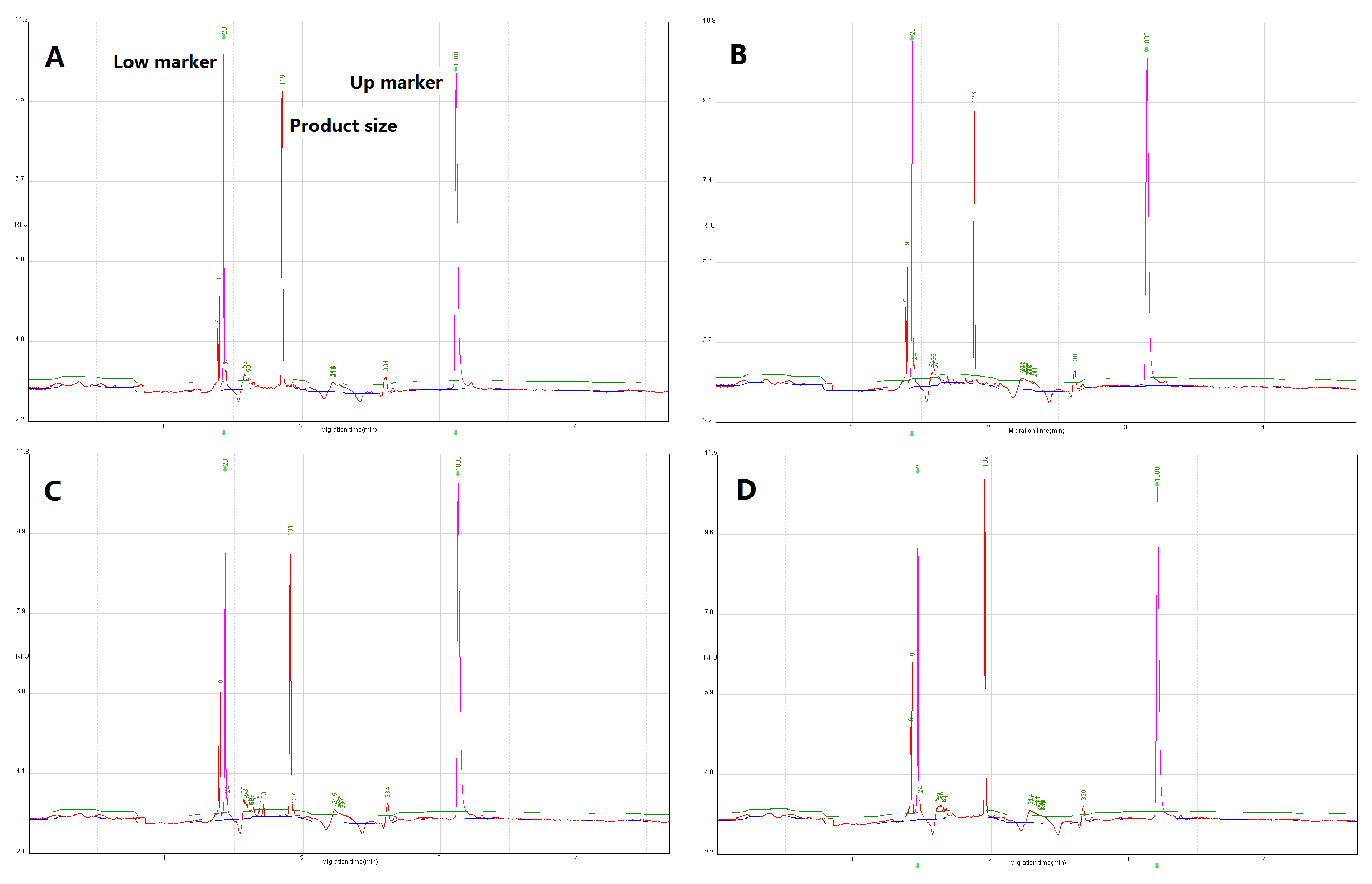

Supplement: Supplementary file 7 — The product sizes of the amplified locus ‘6-73’ in 4 Clematis samples determined by Qsep 100 DNA Analyzer. (PNG 136 kb) [file 41065_2018_60_MOESM7_ESM.png]
